# Advantages and disadvantages of an objective selection process for early intervention in employees at risk for sickness absence

**DOI:** 10.1186/1471-2458-7-67

**Published:** 2007-05-02

**Authors:** Saskia FA Duijts, IJmert Kant, Gerard MH Swaen

**Affiliations:** 1 Maastricht University, Department of Epidemiology, P.O. Box 616, 6200 MD Maastricht, The Netherlands; 2 Dow Chemical Terneuzen, Terneuzen, The Netherlands

## Abstract

**Background:**

It is unclear if objective selection of employees, for an intervention to prevent sickness absence, is more effective than subjective 'personal enlistment'. We hypothesize that objectively selected employees are 'at risk' for sickness absence and eligible to participate in the intervention program.

**Methods:**

The dispatch of 8603 screening instruments forms the starting point of the objective selection process. Different stages of this process, throughout which employees either dropped out or were excluded, were described and compared with the subjective selection process. Characteristics of ineligible and ultimately selected employees, for a randomized trial, were described and quantified using sickness absence data.

**Results:**

Overall response rate on the screening instrument was 42.0%. Response bias was found for the parameters sex and age, but not for sickness absence. Sickness absence was higher in the 'at risk' (N = 212) group (42%) compared to the 'not at risk' (N = 2503) group (25%) (OR 2.17 CI 1.63–2.89; p = 0.000). The selection process ended with the successful inclusion of 151 eligible, i.e. 2% of the approached employees in the trial.

**Conclusion:**

The study shows that objective selection of employees for early intervention is effective. Despite methodological and practical problems, selected employees are actually those at risk for sickness absence, who will probably benefit more from the intervention program than others.

## Background

Sickness absence due to psychosocial health complaints, such as fatigue, burnout and work-family conflicts, represents a major concern in Western societies [1]. In the Netherlands, about one in every three employees seen by their occupational physician are absent from work because of such complaints [2,3]. Reintegration of employees after psychosocial absenteeism causes more difficulties and generally takes longer than return to work following physical problems. Also, the consequences of this type of sickness absence involve enhanced payments and reduced productivity [4]. Thus, successful identification and early intervention with these employees can contribute to the prevention of sickness absence, and should receive profound attention in occupational health practice. Traditionally, intervention programs to reduce psychosocial health complaints, and following sickness absence, are accessible for employees who acknowledge wrestling with these problems. Also, supervisors or personnel departments who notice employees struggling with psychosocial health difficulties support participation in such purposive programs. Both subjective initiatives frequently depend on features such as having certain personality characteristics, holding specific positions or working at particular departments, and are only feasible if financial support from the employer is available and, at best, after repeated sickness absence spells. Overall, work-related intervention programs are initiated when the employee, supervisor or personnel department explicitly summons for assistance, in case of psychosocial health complaints or sickness absence, and if it is within the bounds of possibilities of the company. Though, one could ask oneself if this is the most adequate selection method, when it comes to effectively identifying and preventive intervening. Employees who are aware of an explicit problem and who are able to formulate the underlying issue do not automatically have to be those who benefit most from an intervention program. As well, intervening with those frequently on sick leave does not have to be the timeliest strategy to prevent further sickness absence. Alternatively, intervention programs could be directed at apparently healthy employees, who are objectively selected and identified to be at increased risk for sickness absence due to psychosocial health complaints. These employees may lack insight in their personal situation and may not be able to optimally manage problems that increase their risk on this type of sickness absence. However, certain fundamental prerequisites should be met to make this selection procedure practicable. Specifically, it must be feasible to objectively screen and identify employees at risk for sickness absence, and there must be an effective preventive intervention program. These requisites involve three essential challenges, i.e. a concise screening instrument for early identification of employees must be available; employees must be willing to be screened for their increased risk for sickness absence and selected employees need to be motivated to participate in a non-requested intervention program. In a foregoing study, an instrument consisting of predictive items for both men and women was developed to screen employees for their risk of sickness absence due to psychosocial health complaints [5,6]. With regard to the second and third motivational condition, it is unclear if objective selection is more effective than the traditional subjective selection method of personal enlistment. In this paper, we reported different stages of the objective selection process throughout which employees either dropped out or were excluded, and compared it with the subjective selection process. Also, characteristics of ineligible and ultimately selected employees, for participation in a randomized trial, were described. We hypothesize that these selected employees are actually those at risk for sickness absence, who will consequently benefit more from the preventive intervention program than others. If so, the objective selection process is thought to be effective and successful.

## Methods

### Randomized controlled trial

**Figure 1:**
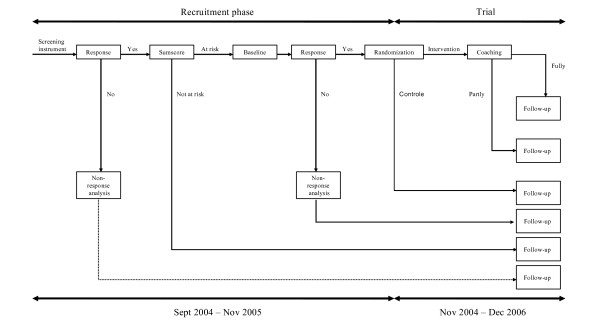
Design of the trial.

The present study is part of a larger randomized trial on the effectiveness of a preventive intervention for employees at risk for sickness absence due to psychosocial health complaints. All employees of participating companies received a screening instrument at their home address. Participating employees received a more extensive baseline questionnaire and were randomized to be included in the trial. Employees allocated to the control group received care as usual, while employees in the intervention group were invited for a preventive coaching program. Follow-up data will be collected until the end of 2006, through self-administered questionnaires. Company records were used to gain insight in duration of sick leave (in days) and frequency of sickness absence (in spells) of each employee. The Medical Ethics Committee of Maastricht University/Academic Hospital Maastricht approved the study protocol. The design of the trial is presented in Figure 1.

### Identification of eligible employees

Three companies, i.e. two universities and a health care centre, situated in the south-eastern part of the Netherlands participated. A total of 8603 employees received an invitational letter, an informative flyer and the screening instrument with informed consent, followed by a reminder two weeks later. The instrument is four pages long with a total of 34 predictive items, 17 for men and 17 for women, completed with a few general demographic items, and exclusion criteria. For both men and women, general health factors, mental health factors, work-related factors, and factors concerning the private situation, were included. Eligibility was assessed through calculation of the individual sum score on each returned screening instrument and verification of the signed informed consent. Employees were 'at risk' for sickness absence if there score was ≥ -3.03 for men and ≥ -2.39 for women. Exclusion criteria were 'fully or partially sick leave', 'suffering from chronic psychological problems', 'having more than one work contract' and 'pregnancy or being on maternity leave' [5]. When an 'at risk' employee returned the baseline questionnaire, signed the second informed consent, and was allocated to the intervention group, an invitational letter with contact details of a specific coach in the geographic area of the employee was dispatched. Two of the participating companies requested that their selected employees contacted the assigned coach, thereby expectantly increasing commitment of the employee to the entire coaching program. One company preferred the coach to contact the selected employees for the first appointment. Also, two companies requested the preventive coaching program to be offered outside the office, whereas the third company favoured the coaching to occur on the premises. Employees allocated to the control group were free to make use of the care present in their company, such as consultation with a social worker or occupational physician.

### Preventive coaching program

The preventive coaching in question is an existing intervention, which consists of seven to nine one-hour sessions within the course of six months. The intervention has been written down in a coaching protocol. The participating coaches received extensive information on the trial and the protocol. They were experienced and specialized in coaching employees at risk for sickness absence, but had to cope with the objective screening of employees as opposed to the more usual personal enlistment. A consequence of this objective screening is that a small number of employees start the preventive coaching program without (being aware of) an explicit problem. This necessitates a somewhat different approach by the coaches in the first introductory interview, i.e. the underlying issue still has to be recognized. Personal and coaching objectives are discussed and an overall question is formulated. At the end of this session, the employee has to be committed to attend the whole preventive coaching program. The second session is a three-way consultation, involving the employee, the involved supervisor and the coach. In this session, objectives from the first consultation are communicated to the supervisor and indispensable organizational objectives are added. The third up to and including the eighth session are individual meetings between employee and coach, in which methodologies and instruments of preventive coaching are applied. The program ends with a second three-way consultation, in which gained insights and experiences are discussed with the supervisor and future plans are made. Qualitative information of each session is registered on checklists, and the coach writes out an overall report of the program.

### Statistical analyses

The χ^2 ^statistic was used to compare overall differences between responding and non-responding employees to the screening instrument. By means of the objective sickness absence data from employer's records, we were able to perform non-response analyses on this primary outcome measure of the trial. Differences between 'at risk' and 'not at risk' employees, regarding sickness absence, were determined. Subsequently, exclusion criteria were applied and dissimilarities between both groups were tested and described by means of usual descriptive analyze techniques. Within the 'at risk' group, the χ^2 ^statistic was used to detect differences between responding and non-responding employees, but now with respect to the baseline questionnaire. Finally, reasons of employees for non-response on the baseline questionnaire or on the invitation for the preventive coaching program were presented. The analyses were performed using the Statistical Package for Social Sciences (SPSS) version 13.0 [7].

## Results

### Non-response screening instrument

**Figure 2:**
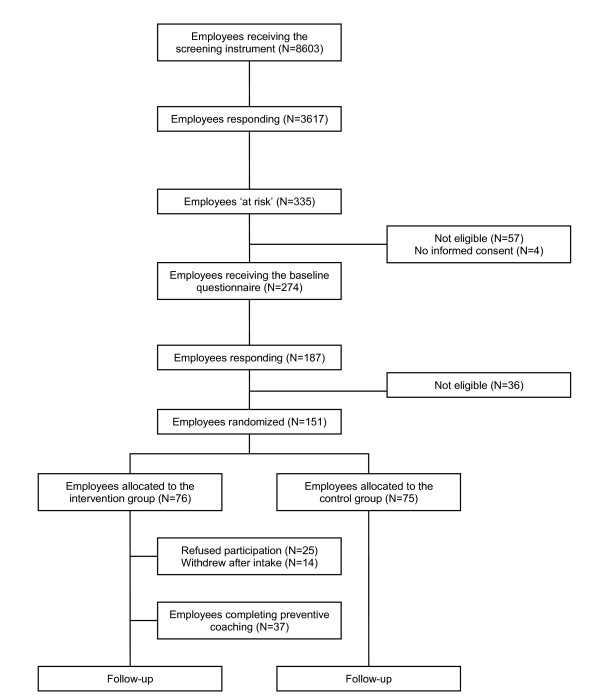
Flowchart of employee selection.

Figure 2 presents the flow of employees through the recruitment phase of the trial. Our database consisted for 58% of employees from universities and for 42% of employees working in the health care sector. The overall response rate on the 8603 screening instruments was 42.0%. As shown in Table 1, the percentage men was lower in the respondents than in the non-respondents. The percentage women was approximately the same in both groups. A χ^2 ^test showed that this difference was significant (p = 0.000). Also, response rates of employees in the intermediate age groups (35–44 and 45–54) were higher than outermost age groups (0–35 and 55–64) (p = 0.000). To explore to what extent sickness absence contributes to a possible response bias, we gathered objective sickness absence data from employer's records of two participating companies (N = 6323). The percentage employees on sick leave in the time of the mailing was lower, but not significant, in the respondents group compared to the non-respondents groups (p = 0.609). Furthermore, the percentage employees (excluding the intervention group) that reported sickness absence in the year after the mailing, was higher, but not significant, in the respondents group compared to the non-respondents group (p = 0.676). As regards duration of sick leave (days) and frequency of sickness absence (spells), no difference was found between the medians of the respondents and non-respondents (p = 0.468 and p = 0.708 respectively) (Table 1).

**Table 1:** Non-response analyses on the screening instrument and the baseline questionnaire

Characteristics	Screening instrument		Baseline questionnaire	
	
	Response (N = 3617)	Non-response (N = 4986)	Response (N = 187)	Non-response (N = 87)
Sex	χ^2 ^= 169.04; p = 0.000		χ^2 ^= 0.00; p = 0.949	
Men	1162 (34%)	2296 (66%)	35 (69%)	16 (31%)
Women	2455 (48%)	2690 (52%)	152 (68%)	71 (32%)
Age	χ^2 ^= 71.36; p = 0.000		χ^2 ^= 4.28; p = 0.233	
0 – 34	1029 (36%)	1841 (64%)	40 (60%)	27 (40%)
35 – 44	1095 (45%)	1320 (55%)	56 (67%)	28 (33%)
45 – 54	1077 (46%)	1262 (54%)	72 (73%)	26 (27%)
55 – 64	416 (42%)	563 (58%)	19 (76%)	6 (24%)
Sickness absence*	Response (N = 2715)	Non-response (N = 3545)	Response (N = 94)	Non-response (N = 71)
Yes/no	χ^2 ^= 0.175; p = 0.676		χ^2 ^= 0.140; p = 0.708	
Duration (in days)	p = 0.468		p = 0.926	
Frequency (in spells)	p = 0.708		p = 0.347	

* Sickness absence data of only two companies were available.

### At risk versus not at risk

From the respondents on the screening instrument (N = 3617), 335 employees were identified as being 'at risk' for sickness absence due to psychosocial health complaints. 'At risk' employees from two companies providing sickness absence data, excluding the intervention group, (N = 212) were compared to the 'not at risk' employees (N = 2503). The percentage employees that reported sickness absence (one or more times in the last four months) was higher in the 'at risk' group (42%) compared to the 'not at risk' group (25%) (OR 2.17 CI 1.63–2.89; p = 0.000). For the duration of sick leave (days) and frequency of sickness absence (spells), significant differences were found between the 'at risk' and 'not at risk' groups (p = 0.000 and p = 0.000 respectively). Exclusion criteria were applied to all respondents (N = 3617) on the screening instrument to explore dissimilarities between the complete 'at risk' group (N = 335; exclusion 18.2%) and 'not at risk' group (N = 3282; exclusion 16.2%), regarding these specific criteria. The percentage of employees fully or partially on sick leave was 17.0% in the 'at risk' group and 5.3% in the 'not at risk' group (p = 0.000). The percentage of employees reporting to suffer from chronic psychological problems at baseline was 14.0% in the 'at risk' group and 1.6% in the 'not at risk' group (p = 0.000). The percentage of employees that had more than one contract was 11.3% in the 'at risk' group and 8.2% in the 'not at risk' group (p = 0.052). Finally, the percentage of employees that was pregnant or on maternity leave was 0.9% in the 'at risk' group and 1.5% in the 'not at risk' group (p = 0.382).

### Non-response baseline questionnaire

The baseline questionnaire was sent to the remaining 'at risk' employees (N = 274). In accordance with the non-response analyses on the screening instrument, we repeated these analyses to detect differences between responding (N = 187) and non-responding employees (N = 87) on the baseline questionnaire. As shown in Table 1, the analysis revealed no significant differences between respondents and non-respondents with respect to sex (p = 0.949) and age (p = 0.233). The percentage of participating employees reporting sickness absence in the year after the baseline questionnaire was sent, was lower in the respondents group compared to the non-respondents group. A χ^2 ^test showed that this difference was not significant (p = 0.708). For duration of sick leave (days) and frequency of sickness absence (spells), no difference was found between the respondents and non-respondents. Results for both tests were not significant (p = 0.926 and p = 0.374 respectively) (Table 1).

### Reasons for non-response

The main reasons for non-response on the baseline questionnaire (N = 87) were 'personal matters' (61.0%), such as 'I don't feel like it' (19.1%) or 'I can deal with these problems myself' (17.1%); 'issues regarding the preventive coaching program' (39.0%), such as 'I don't want an invitation for the preventive coaching program' (19.5%) or 'I don't want my supervisor to participate in the coaching program' (12.2%); 'organizational matters' (29.3%), such as 'I am afraid of consequences caused by participation' (12.2%); and 'other reasons' (31.7%), such as 'I have lost the questionnaire' (12.2%). Employees who reported to be on sick leave at the time of the baseline mailing, were excluded from the study (N = 36). As a result, 151 'at risk' employees were left for random allocation to receive the preventive coaching program (N = 76) or 'care as usual' (N = 75). The main reasons for declining the invitation for the preventive coaching program (N = 25) were 'personal matters' (64.3%), such as 'I can deal with these problems myself' (28.6%) or 'I don't understand why I received this invitation' (14.3%); 'issues regarding the preventive coaching program' (35.7%), such as 'I don't want my supervisor to participate in the coaching program' (14.3%); and finally, 'organizational matters' (35.7%), such as 'I am too busy at work to participate in the coaching program' (28.6%). Eventually, 51 employees entered the preventive coaching program through this objective selection process.

## Discussion

### General findings

This study describes the objective selection process foregoing a preventive intervention for employees at risk for sickness absence due to psychosocial health complaints. Because of the successive stages and various components in this procedure, such as the response to questionnaires, the determination of being 'at risk' for sickness absence, the multi-stage informed consent and the acceptance of the invitation for the preventive coaching program, the enrolment of eligible employees was a comprehensive task. Keeping Lasagna's Law [8] in mind, stating that no reliable enrolment estimates can be made, the selection phase of this study started with the dispatch of 8603 screening instruments and ended with the inclusion of 151 employees in the trial. Notwithstanding this seemingly successful recruitment phase, we initially expected a substantially larger number of employees, allocated to the intervention group. Nevertheless, the overall selection phase yielded more significant information than anticipated. We will interpret the eligibility of employees throughout the successive stages of the objective selection process, present limitations, and give recommendations for future research and implementation possibilities.

### Eligibility of employees

#### Non-response

The screening instrument was mailed to the home addresses of employees of three large companies to identify employees 'at risk' for sickness absence due to psychosocial health complaints. Non-response to mailed questionnaires reduces the effective sample size and can introduce bias [9]. We used our premeditated sample of 150 employees as starting point and estimated the total amount of screening instruments to be dispatched, i.e. a minimum of 8000. Hereby, we attempted to account for circumstances such as non-response of approximately 50% on the screening instrument, exclusion of about 20% of responding employees, estimated prevalence of 10% 'at risk' employees for sickness absence, non-response of 40% on the baseline questionnaire and exclusion of about 20% of responding employees on the baseline questionnaire. These percentages were based on experiences from previous research [6]. Further, bias may be introduced if non-responders differ significantly, particularly regarding sickness absence, from those who do respond. Therefore, it is important to examine the characteristics of responders and non-responders to determine the degree to which the sample may be said to be representative of the population. This will permit the generalization to the population, or indicate the degree of caution needed [10]. Since significant differences were found in sex and age, the results of our upcoming trial have to be corrected for or stratified on these specific characteristics. No difference was found in sickness absence between responding and non-responding employees. Notwithstanding this positive outcome, there is no denying that objective selection has the consequence that many eligible employees at risk for sickness absence can not be included in the preventive intervention, due to their non-response on the screening instrument.

#### Not at risk

To assure that the intervention will be directed at employees who will particularly benefit from it and to restrict the number of false-positively classified healthy working employees, we preferred a screening instrument with a high specificity [5]. As a result, numerous employees were assigned to the 'not at risk' group, hereby once more reducing the number of employees eligible for randomization. Still, information on these ineligible employees, specifically regarding sickness absence, is extremely useful as it will enable us to externally validate the screening instrument. And, the significant difference in sickness absence between 'at risk' and 'not at risk' employees supports the fact that principally eligible employees were selected through objective selection.

#### Exclusion

Exclusion criteria were applied to both 'at risk' and 'not at risk' employees to get an initial impression of the instrument characteristics. The percentage of employees to be excluded from the 'at risk' group turned out to be twice the percentage of employees to be excluded from the 'not at risk' group. The criteria 'being fully or partially on sick leave' and 'suffering from chronic psychological problems at baseline' were the most imperative, statistically significant reasons for this difference. The provisionally presumption can be made that eligible employees, i.e. those at risk for sickness absence, can be reasonably identified using the screening instrument. However, in order to be able to pronounce upon the generalization to the population, external validation of the screening instrument and insight into additional psychometric characteristics of the instrument are indispensable.

#### Withdrawal

All employees eligible for the trial received the baseline questionnaire and a second informed consent. No differences were found in sickness absence between respondents and non respondents on this questionnaire. In order to retain a sufficient number of eligible employees, it was necessary to meet a few conditions. Firstly, a high response rate on the baseline questionnaire was pursued by giving the selected employees comprehensive information on the trial, sending them two reminders and an extra questionnaire in the following month, and by retrieving reasons for non-response in case of definite disinterest. Secondly, employees were acquainted with the possible control group allocation and were free to choose 'care as usual' or additional treatment, if necessary. Thirdly, participating companies were free to choose the method of contact between coach and employee and the location of the coaching program, in order to attune the program on their employees and the standards of the company. Finally, the multi-stage informed consent procedure was chosen to thoroughly inform the employees on the consequences of partaking, as preventive coaching implies an intensive commitment. With this, we expected to reduce the number of employees, allocated to the intervention group, declining the invite for the preventive coaching program. Still, a third of the invited employees refused to participate in the preventive coaching program. The main reasons for non-response were related to 'personal matters' and 'issues related to the preventive coaching program' in both non-responders to the baseline questionnaire and non-responders to the coaching program. For example, employees preferred coping with the identified problems by themselves. Probably, employees would be more receptive for support in managing their problems when easy accessible service is continuously available on the premises. Also, employees expressed concern about the participation of their supervisor in the preventive coaching program. Apparently, employees prefer to avoid this confrontation in a three-way consultation. It is plausible that an intervention without such a confrontation is more attractive and, accordingly, has an effect on the number of employees agreeing to randomization. Nevertheless, the spearhead of the preventive coaching program is its reflection to the organization and the input of the supervisor in this matter can not be denied. Eventually, about 2% of the initially approached employees passed through every described stage of the objective selection process, and entered our trial on the effectiveness of a preventive coaching program for sickness absence.

### Comparison of objective and subjective selection

Keeping the hypothesis of this study in mind, one can ask oneself if this objective selection process is in fact more effective than the traditional subjective selection method of personal enlistment. Imperative issues, such as non-response, have to be considered when objectively identifying employees to be at risk for sickness absence. Though, such issues are also significant for the subjective route. For instance, the former can be directed at all employees of a company and has the advantage of neutral allocation of the intervention program, specifically for employees who do not let it be known to struggle with psychosocial health complaints. The latter implicitly gives precedence to the employees who monopolize this opportunity by simply asking for participation or informing their supervisor about their problems. Through this, participation in purposive intervention programs is depending on the degree of articulation and personal awareness of the employee. However, this subjective personal enlistment of employees does not imply that they are actually 'at risk' for sickness absence, and by that belong to the target group of the specific intervention program. Therefore, the definition of the intended target group is essential in deciding between objective and subjective procedures. Further, the objective selection method has to contend with non-response on questionnaires and non-commitment to the intervention program. Several employees, who would almost certainly benefit from the intervention, may stay unidentified this way. In contrast, the subjective method starts from the idea that partaking is a self-imposed task, and consequently has the advantage of minimum selective withdrawal. Still, the former is not automatically put at a disadvantage, since requisites of partaking in an intervention program, such as commitment, are probably not unconditionally present in the non-responders. Unpremeditated exclusion of these employees could positively influence the effect of the intervention program. Before putting a selection method into practice, one should determine if preventive or curative intervening is indicative. When the objective of a company is to independently identify employees for the prevention of sickness absence, the only possible selection method is objective identification of eligible employees.

## Conclusion

This study shows that objective selection to enable preventive intervention is effective and successful. Selected employees are actually those at risk for sickness absence, who will probably benefit more from the preventive intervention program than others. However, methodological problems, such as non-response, are undeniable and the whole process is expensive and time-consuming. Using the subjective method of personal enlistment, the eligibility of employees is not definite, though abovementioned problems are faced to a lesser degree. Thus, before putting a selection method into practice, employees, supervisors or personnel departments should determine which objectives should be given priority, and they should understand the consequences of all stages of the selection process. Interpretation of the presented findings must be seen within the framework of our trial. Nevertheless, our experiences and quantitative information on the effectiveness of the process can prompt researchers to prevent and overcome difficulties mentioned here in future.

## Competing interests

The author(s) declare that they have no competing interests.

## Authors' contributions

All authors (1) have made substantial contributions to conception and design of this study, data analysis, and interpretation of data; (2) have been involved in drafting the manuscript or revising it critically for important intellectual content; (3) and have given final approval of the version to be published.

## Pre-publication history

The pre-publication history for this paper can be accessed here:


